# Cinnarizinium dipicrate

**DOI:** 10.1107/S1600536811002674

**Published:** 2011-01-26

**Authors:** Jerry P. Jasinski, Ray J. Butcher, M. S. Siddegowda, H. S. Yathirajan, C. S. Chidan Kumar

**Affiliations:** a Department of Chemistry, Keene State College, 229 Main Street, Keene, NH 03435-2001, USA; b Department of Chemistry, Howard University, 525 College Street NW, Washington, DC 20059, USA; c Department of Studies in Chemistry, University of Mysore, Manasagangotri, Mysore 570 006, India

## Abstract

In the cinnarizinium dication of the title compound {systematic name: 1-diphenyl­methyl-4-[(2*E*)-3-phenyl­prop-2-en-1-yl]piperazine-1,4-diium bis­(2,4,6-trinitro­phenolate)}, C_26_H_30_N_2_^2+^·2C_6_H_2_N_3_O_7_^−^, the piperazine group is protonated at both N atoms and adopts a slightly distorted chair conformation. Strong N—H⋯O_hy­droxy_ cation–anion hydrogen bonds link the dication and two anions. In the cation, the (2*E*)-3-phenyl­prop-2-en-1-yl fragment is disordered over two positions in a ratio of 0.586 (4): 0.414 (4). Two nitro groups in one anion and three in the other one demonstrate rotational disorder. The crystal packing is stabilized by weak inter­molecular π–π [centroid–centroid distances = 3.844 (7), 3.677 (9), 3.825 (5), 3.634 (2) and 3.729 (7) Å], C—H⋯π and C—H⋯O inter­actions.

## Related literature

For background to the anti­histamine cinnarizine (systematic name: 1-benzhydryl-4-cinnamyl-piperazine), see: Barrett & Zolov (1960 ▶); Towse (1980 ▶). For the structure of opipramol dipicrate {systematic name: 1-[3-(5*H*-dibenz[*b*,*f*]azepin-5-yl)prop­yl]-4-(2-hy­droxy­eth­yl)piperazine-1,4-diium bis­(2,4,6-tri­nitro­phrenolate)}, see: Jasinski *et al.* (2010 ▶). For related structures, see: Bertolasi *et al.* (1980 ▶); Mouillé *et al.* (1975 ▶). For puckering parameters, see: Cremer & Pople (1975 ▶). For standard bond lengths, see: Allen *et al.* (1987 ▶).
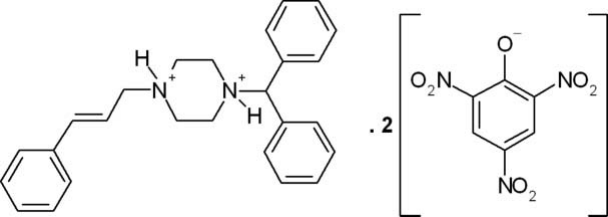


## Experimental

### 

#### Crystal data

C_26_H_30_N_2_^2+^·2C_6_H_2_N_3_O_7_^−^
*M* *_r_* = 826.73
Monoclinic, 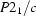

*a* = 15.1987 (2) Å
*b* = 10.09130 (17) Å
*c* = 25.0724 (3) Å
β = 95.9170 (14)°
*V* = 3824.98 (10) Å^3^
*Z* = 4
Cu *K*α radiation
μ = 0.95 mm^−1^
*T* = 295 K
0.49 × 0.42 × 0.27 mm

#### Data collection

Oxford Diffraction Xcalibur Ruby Gemini diffractometer
Absorption correction: multi-scan (*CrysAlis RED*; Oxford Diffraction, 2007 ▶) *T*_min_ = 0.777, *T*_max_ = 1.000
14786 measured reflections
7319 independent reflections
5784 reflections with *I* > 2σ(*I*)
*R*_int_ = 0.024

#### Refinement

*R*[*F* ^2^ > 2σ(*F* ^2^)] = 0.092
*wR*(*F* ^2^) = 0.289
*S* = 1.01
7319 reflections
648 parameters
40 restraints
H atoms treated by a mixture of independent and constrained refinement
Δρ_max_ = 1.24 e Å^−3^
Δρ_min_ = −0.48 e Å^−3^

### 

Data collection: *CrysAlis PRO* (Oxford Diffraction, 2007 ▶); cell refinement: *CrysAlis PRO*; data reduction: *CrysAlis RED* (Oxford Diffraction, 2007 ▶); program(s) used to solve structure: *SHELXS97* (Sheldrick, 2008 ▶); program(s) used to refine structure: *SHELXL97* (Sheldrick, 2008 ▶); molecular graphics: *SHELXTL* (Sheldrick, 2008 ▶); software used to prepare material for publication: *SHELXTL*.

## Supplementary Material

Crystal structure: contains datablocks global, I. DOI: 10.1107/S1600536811002674/cv5030sup1.cif

Structure factors: contains datablocks I. DOI: 10.1107/S1600536811002674/cv5030Isup2.hkl

Additional supplementary materials:  crystallographic information; 3D view; checkCIF report

## Figures and Tables

**Table 1:** Hydrogen-bond geometry (Å, °) *Cg*5 and *Cg*6 are the centroids of the C6–C11 and C12–C17 rings, respectively.

*D*—H⋯*A*	*D*—H	H⋯*A*	*D*⋯*A*	*D*—H⋯*A*
N1—H1*N*⋯O1*A*	0.90 (1)	1.77 (1)	2.658 (3)	168 (3)
N2—H2*N*⋯O1*B*	0.90 (1)	1.83 (3)	2.603 (3)	143 (4)
N2—H2*N*⋯O7*BA*	0.90 (1)	2.30 (3)	3.047 (5)	140 (3)
C3—H3*A*⋯O2*AA*	0.97	2.50	3.214 (8)	130
C3—H3*A*⋯O2*AB*	0.97	2.52	3.242 (17)	131
C2—H2*B*⋯O7*AA*	0.97	2.48	3.304 (5)	142
C2—H2*B*⋯O7*AB*	0.97	2.54	3.42 (4)	151
C11—H11*A*⋯O4*BA*^i^	0.93	2.57	3.244 (4)	130
C17—H17*A*⋯O1*A*	0.93	2.62	3.473 (4)	154
C17—H17*A*⋯O5*BB*^i^	0.93	2.52	3.267 (8)	138
C18*A*—H18*A*⋯*Cg*6^ii^	0.97	2.88	3.742 (5)	148
C18*A*—H18*B*⋯*Cg*5^ii^	0.97	2.83	3.762 (1)	161
C18*A*—H18*C*⋯*Cg*5^ii^	0.97	3.00	3.762 (1)	137
C18*A*—H18*D*⋯*Cg*6^ii^	0.97	2.84	3.742 (5)	155

Symmetry codes: (i) 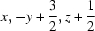; (ii) 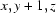.

**Fig. 1.**
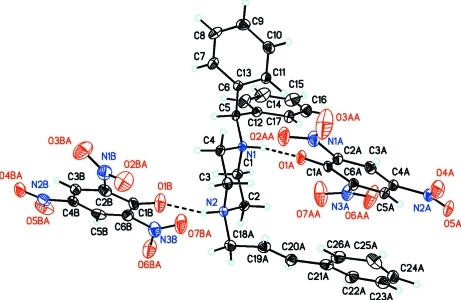
Molecular structure of the title compound showing the atom labeling scheme and 30% probability displacement ellipsoids. Dashed lines indicate strong intermolecular N—H···O hydrogen bonds between the protonated N atoms from the piperazine group in the cinnarizinium cation and the two picrate anions. For the disordered atoms, only major components are shown.

**Fig. 2.**
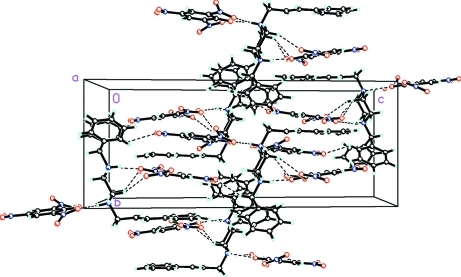
Packing diagram of the title compound viewed down the a axis. Dashed lines indicate N—H···O hydrogen bonds and weak C—H···O intermolecular interactions creating a 2-D network structure.

## supplementary crystallographic information

### Comment

Cinnarizine is an antihistamine which is mainly used for the control of nausea and vomiting due to motion sickness. Cinnarizine could be also viewed as a nootropic drug because of its vasorelaxating abilities (due to calcium channel blockage), which happen mostly in brain and is also used as a labyrinthine sedative (Towse, 1980). A clinical evaluation of cinnarizine in various allergic disorders is published (Barrett & Zolov, 1960). Cinnarizine can be used in scuba divers without an increased risk of central nervous system oxygen toxicity. The crystal structures of some related compounds *viz*., cinnarizine (Mouillé *et al.*, 1975) and cyclizine hydrochloride (Bertolasi *et al.*, 1980) have been reported. In continuation of our work on the picrates of pharmaceutical compounds, we have recently reported the crystal structure of opipramol dipicrate (Jasinski *et al.*, 2010). In view of the importance of cinnarizine, this paper reports the crystal structure of the title compound, C_26_H_30_N_2_^2+^. 2(C_6_H_2_N_3_O_7_)^-^, (I).

In the cinnarizinium dication of the title compound, (I), [systematic name: 1-benzhydryl-4-cinnamyl-piperazine dipicrate] the piperazine group is protonated at both N atoms and adopts a slightly distorted chair conformation with puckering parameters Q, θ and φ of 0.58 (3) Å, 177.1 (0)° and 175.518 (5)° (Cremer & Pople, 1975) (Fig. 1). For an ideal chair θ has a value of 0 or 180°. The dihedral angle between the mean planes of the cation piperazine ring and three benzene rings or the six-membered rings of the picrate anions are 75.9 (6)° 80.0 (2)°, 80.4 (9)° or 71.4 (9)° and 86.7 (6)°, respectively. Bond distances (Allen *et al.*, 1987) and angles are in normal ranges. Strong *N*—*H*···O_hydroxy_ cation-anion hydrogen bonds link the dication and two anions. In the cation, the (2E)-3-phenylprop-2-ene-1yl fragment is disordered over two positions in a ratio of 0.586 (4): 0.414 (4). Two nitro groups in one anion (A) and three in the other (B) demonstrate rotational disorder [O2AA & O3AA (0.68 (2)), O2AB & O3AB (0.32 (2)); O6AA & O7AA (0.851 (14)), O6AB & O7AB (0.149 (14)); O2BA & O3BA (0.80 (2)), O2BB & O3BB (0.20 (2)); O4BA & O5BB (0.951 (19)), O4BB & O5BB (0.050 (19)); O6BA & O7BA (0.756 (8)), O6BB & O7BB (0.244 (8))]. The crystal packing is stabilized by π–π (Table 1),  C—H···π-ring and C—H···O  (Table 2) weak intermolecular interactions creating a 2-D network structure (Fig. 2).

### Experimental

Cinnarizine (3.68 g, 0.01 mol) and picric acid (2.99 g, 0.01 mol) were dissolved in hot acetonitrile and dimethyl sulphoxide (80:20 *v*/*v*) solution and stirred over a heating magnetic stirrer for few minutes (330 K). The resulting solution was allowed to cool slowly at room temperature. X-ray quality crystals of the title compound appeared after a few days. (M.P.: 481– 483 K).

### Refinement

Carbon atoms C18–C26 are disordered (0.586 (4) A: 0.414 (4) B). The oxygen atoms on the N1A, N3A, N1B, N2B and N3B anion nitro groups are rotationally disordered [O2AA & O3AA (0.68 (2)), O2AB & O3AB (0.32 (2)); O6AA & O7AA (0.851 (14)), O6AB & O7AB (0.149 (14)); O2BA & O3BA (0.80 (2)), O2BB & O3BB (0.20 (2)); O4BA & O5BB (0.951 (19)), O4BB & O5BB (0.050 (19)); O6BA & O7BA (0.756 (8)), O6BB & O7BB (0.244 (8))] H1N and H2N were refined isotropically. All of the remaining H atoms were placed in their calculated positions and then refined using the riding model with Atom—H lengths of 0.93 & 0.98Å (CH), or 0.97Å (CH_2_). Isotropic displacement parameters for these atoms were set to 1.19–1.20 (CH, CH_2_) times *U*_eq_ of the parent atom. The highest residual peak of 1.24 eÅ^-3^ is situated 1.16 Å from atom O1B.

### Crystal data

**Table:**

C_26_H_30_N_2_^2+^·2C_6_H_2_N_3_O_7_^−^	*F*(000) = 1720
*M**_r_* = 826.73	*D*_x_ = 1.436 Mg m^−^^3^
Monoclinic, *P*2_1_/*c*	Cu *K*α radiation, λ = 1.54178 Å
Hall symbol: -P 2ybc	Cell parameters from 7661 reflections
*a* = 15.1987 (2) Å	θ = 4.8–73.3°
*b* = 10.09130 (17) Å	µ = 0.95 mm^−^^1^
*c* = 25.0724 (3) Å	*T* = 295 K
β = 95.9170 (14)°	Chunk, yellow
*V* = 3824.98 (10) Å^3^	0.49 × 0.42 × 0.27 mm
*Z* = 4	

### Data collection

**Table:**

Oxford Diffraction Xcalibur Ruby Gemini diffractometer	7319 independent reflections
Radiation source: Enhance (Cu) X-ray Source	5784 reflections with *I* > 2σ(*I*)
graphite	*R*_int_ = 0.024
Detector resolution: 10.5081 pixels mm^-1^	θ_max_ = 73.5°, θ_min_ = 4.8°
ω scans	*h* = −18→17
Absorption correction: multi-scan (*CrysAlis RED*; Oxford Diffraction, 2007)	*k* = −11→12
*T*_min_ = 0.777, *T*_max_ = 1.000	*l* = −31→20
14786 measured reflections	

### Refinement

**Table:**

Refinement on *F*^2^	Secondary atom site location: difference Fourier map
Least-squares matrix: full	Hydrogen site location: inferred from neighbouring sites
*R*[*F*^2^ > 2σ(*F*^2^)] = 0.092	H atoms treated by a mixture of independent and constrained refinement
*wR*(*F*^2^) = 0.289	*w* = 1/[σ^2^(*F*_o_^2^) + (0.196*P*)^2^ + 2.2706*P*] where *P* = (*F*_o_^2^ + 2*F*_c_^2^)/3
*S* = 1.01	(Δ/σ)_max_ = 0.007
7319 reflections	Δρ_max_ = 1.24 e Å^−^^3^
648 parameters	Δρ_min_ = −0.48 e Å^−^^3^
40 restraints	Extinction correction: *SHELXL97* (Sheldrick, 2008), Fc^*^=kFc[1+0.001xFc^2^λ^3^/sin(2θ)]^-1/4^
Primary atom site location: structure-invariant direct methods	Extinction coefficient: 0.0017 (4)

### Special details

**Table:**

Geometry. All e.s.d.'s (except the e.s.d. in the dihedral angle between two l.s. planes) are estimated using the full covariance matrix. The cell e.s.d.'s are taken into account individually in the estimation of e.s.d.'s in distances, angles and torsion angles; correlations between e.s.d.'s in cell parameters are only used when they are defined by crystal symmetry. An approximate (isotropic) treatment of cell e.s.d.'s is used for estimating e.s.d.'s involving l.s. planes.
Refinement. Refinement of *F*^2^ against ALL reflections. The weighted *R*-factor *wR* and goodness of fit *S* are based on *F*^2^, conventional *R*-factors *R* are based on *F*, with *F* set to zero for negative *F*^2^. The threshold expression of *F*^2^ > σ(*F*^2^) is used only for calculating *R*-factors(gt) *etc*. and is not relevant to the choice of reflections for refinement. *R*-factors based on *F*^2^ are statistically about twice as large as those based on *F*, and *R*- factors based on ALL data will be even larger.

### Fractional atomic coordinates and isotropic or equivalent isotropic displacement parameters (Å^2^)

**Table:**

	*x*	*y*	*z*	*U*_iso_*/*U*_eq_	Occ. (<1)
N1	0.75982 (13)	0.8086 (2)	0.55675 (7)	0.0344 (5)	
H1N	0.759 (2)	0.816 (3)	0.5925 (3)	0.044 (8)*	
N2	0.75781 (15)	0.5191 (2)	0.55806 (8)	0.0413 (5)	
H2N	0.766 (3)	0.501 (4)	0.5239 (5)	0.062 (10)*	
C1	0.84038 (16)	0.7281 (3)	0.54794 (11)	0.0408 (6)	
H1A	0.8932	0.7766	0.5613	0.049*	
H1B	0.8422	0.7134	0.5098	0.049*	
C2	0.83881 (18)	0.5960 (3)	0.57647 (11)	0.0433 (6)	
H2A	0.8907	0.5451	0.5697	0.052*	
H2B	0.8410	0.6109	0.6148	0.052*	
C3	0.67708 (17)	0.5985 (3)	0.56498 (11)	0.0431 (6)	
H3A	0.6732	0.6136	0.6029	0.052*	
H3B	0.6250	0.5492	0.5508	0.052*	
C4	0.67904 (16)	0.7304 (3)	0.53629 (10)	0.0403 (6)	
H4A	0.6792	0.7154	0.4981	0.048*	
H4B	0.6263	0.7806	0.5419	0.048*	
C5	0.76219 (17)	0.9422 (3)	0.52874 (9)	0.0384 (6)	
H5A	0.7662	0.9246	0.4906	0.046*	
C6	0.67862 (17)	1.0214 (3)	0.53265 (10)	0.0394 (6)	
C7	0.6353 (2)	1.0759 (3)	0.48662 (12)	0.0526 (7)	
H7A	0.6541	1.0553	0.4535	0.063*	
C8	0.5645 (2)	1.1607 (4)	0.48926 (15)	0.0671 (9)	
H8A	0.5364	1.1973	0.4580	0.080*	
C9	0.5356 (2)	1.1908 (4)	0.53768 (15)	0.0618 (9)	
H9A	0.4888	1.2494	0.5395	0.074*	
C10	0.5762 (2)	1.1342 (4)	0.58369 (14)	0.0582 (8)	
H10A	0.5551	1.1519	0.6165	0.070*	
C11	0.6478 (2)	1.0515 (3)	0.58150 (11)	0.0497 (7)	
H11A	0.6757	1.0154	0.6129	0.060*	
C12	0.84287 (18)	1.0223 (3)	0.54911 (11)	0.0425 (6)	
C13	0.8878 (2)	1.0922 (4)	0.51268 (15)	0.0679 (10)	
H13A	0.8719	1.0812	0.4761	0.081*	
C14	0.9559 (3)	1.1782 (5)	0.5300 (2)	0.0871 (14)	
H14A	0.9843	1.2259	0.5051	0.105*	
C15	0.9817 (2)	1.1931 (5)	0.5837 (2)	0.0811 (13)	
H15A	1.0276	1.2504	0.5952	0.097*	
C16	0.9390 (2)	1.1228 (4)	0.62025 (16)	0.0675 (9)	
H16A	0.9571	1.1314	0.6567	0.081*	
C17	0.8696 (2)	1.0395 (3)	0.60355 (12)	0.0524 (7)	
H17A	0.8403	0.9944	0.6288	0.063*	
C18A	0.7558 (2)	0.3854 (3)	0.58610 (13)	0.0561 (8)	0.586 (4)
H18A	0.8131	0.3431	0.5858	0.067*	0.586 (4)
H18B	0.7122	0.3292	0.5662	0.067*	0.586 (4)
C19A	0.7334 (4)	0.3969 (6)	0.6447 (3)	0.0529 (11)	0.586 (4)
H19A	0.6748	0.4071	0.6515	0.063*	0.586 (4)
C20A	0.7955 (4)	0.3926 (5)	0.6849 (2)	0.0520 (10)	0.586 (4)
H20A	0.8529	0.3849	0.6755	0.062*	0.586 (4)
C21A	0.7875 (8)	0.3981 (12)	0.7433 (4)	0.053 (3)	0.586 (4)
C22A	0.8612 (7)	0.3990 (12)	0.7805 (5)	0.058 (2)	0.586 (4)
H22A	0.9177	0.4000	0.7693	0.070*	0.586 (4)
C23A	0.8507 (14)	0.398 (2)	0.8307 (10)	0.066 (5)	0.586 (4)
H23A	0.9011	0.3996	0.8551	0.079*	0.586 (4)
C24A	0.7726 (14)	0.3958 (14)	0.8500 (8)	0.068 (7)	0.586 (4)
H24A	0.7739	0.3856	0.8869	0.082*	0.586 (4)
C25A	0.691 (2)	0.406 (3)	0.8224 (14)	0.079 (8)	0.586 (4)
H25A	0.6372	0.4152	0.8369	0.094*	0.586 (4)
C26A	0.7035 (14)	0.401 (3)	0.7669 (14)	0.072 (5)	0.586 (4)
H26A	0.6525	0.3995	0.7430	0.086*	0.586 (4)
C18B	0.7558 (2)	0.3854 (3)	0.58610 (13)	0.0561 (8)	0.414 (4)
H18C	0.6960	0.3507	0.5815	0.067*	0.414 (4)
H18D	0.7936	0.3238	0.5693	0.067*	0.414 (4)
C19B	0.7860 (6)	0.3939 (8)	0.6445 (4)	0.0529 (11)	0.414 (4)
H19B	0.8460	0.3997	0.6561	0.063*	0.414 (4)
C20B	0.7274 (6)	0.3932 (8)	0.6793 (3)	0.0520 (10)	0.414 (4)
H20B	0.6679	0.3888	0.6663	0.062*	0.414 (4)
C21B	0.7502 (10)	0.3992 (16)	0.7386 (4)	0.044 (3)	0.414 (4)
C22B	0.8333 (9)	0.3969 (16)	0.7670 (6)	0.051 (3)	0.414 (4)
H22B	0.8837	0.3942	0.7489	0.061*	0.414 (4)
C23B	0.841 (3)	0.399 (3)	0.826 (2)	0.104 (14)	0.414 (4)
H23B	0.8975	0.3976	0.8442	0.125*	0.414 (4)
C24B	0.771 (2)	0.402 (2)	0.8544 (11)	0.071 (10)	0.414 (4)
H24B	0.7724	0.4088	0.8915	0.085*	0.414 (4)
C25B	0.694 (3)	0.395 (3)	0.8183 (13)	0.050 (5)	0.414 (4)
H25B	0.6429	0.3848	0.8351	0.060*	0.414 (4)
C26B	0.6787 (18)	0.400 (3)	0.7629 (13)	0.052 (4)	0.414 (4)
H26B	0.6222	0.4029	0.7447	0.062*	0.414 (4)
O1A	0.7492 (2)	0.7961 (3)	0.66177 (8)	0.0704 (7)	
O2AA	0.5803 (8)	0.7737 (14)	0.64870 (13)	0.108 (3)	0.68 (2)
O3AA	0.5075 (5)	0.803 (2)	0.7157 (4)	0.169 (5)	0.68 (2)
O2AB	0.5627 (18)	0.742 (3)	0.6507 (4)	0.108 (3)	0.32 (2)
O3AB	0.5305 (13)	0.863 (2)	0.7152 (11)	0.169 (5)	0.32 (2)
O4A	0.64470 (17)	0.7000 (3)	0.89225 (10)	0.0867 (9)	
O5A	0.78481 (17)	0.7067 (3)	0.90778 (9)	0.0809 (9)	
O6AA	0.9618 (2)	0.7918 (10)	0.76971 (16)	0.134 (3)	0.851 (14)
O7AA	0.9173 (3)	0.7450 (11)	0.68875 (15)	0.143 (3)	0.851 (14)
O6AB	0.9535 (15)	0.851 (2)	0.7519 (13)	0.134 (3)	0.149 (14)
O7AB	0.924 (2)	0.680 (4)	0.7036 (18)	0.143 (3)	0.149 (14)
N1A	0.57602 (18)	0.7801 (4)	0.69661 (10)	0.0703 (8)	
N2A	0.71761 (14)	0.7114 (3)	0.87725 (8)	0.0593 (7)	
N3A	0.90351 (17)	0.7638 (4)	0.73487 (10)	0.0758 (9)	
C1A	0.7410 (2)	0.7722 (3)	0.70954 (10)	0.0431 (6)	
C2A	0.6581 (2)	0.7630 (3)	0.73172 (11)	0.0462 (6)	
C3A	0.6493 (2)	0.7436 (3)	0.78570 (12)	0.0493 (7)	
H3AA	0.5936	0.7396	0.7979	0.059*	
C4A	0.7242 (2)	0.7305 (3)	0.82064 (10)	0.0453 (6)	
C5A	0.8072 (2)	0.7370 (3)	0.80328 (11)	0.0478 (7)	
H5AA	0.8574	0.7286	0.8276	0.057*	
C6A	0.8152 (2)	0.7560 (3)	0.74985 (11)	0.0474 (7)	
O1B	0.7062 (2)	0.4822 (4)	0.45690 (11)	0.0924 (10)	
O2BA	0.5305 (6)	0.4599 (7)	0.4261 (2)	0.1086 (19)	0.80 (2)
O3BA	0.5020 (6)	0.5403 (14)	0.3479 (3)	0.126 (2)	0.80 (2)
O2BB	0.5629 (15)	0.483 (4)	0.43148 (19)	0.1086 (19)	0.20 (2)
O3BB	0.493 (3)	0.556 (6)	0.3597 (9)	0.126 (2)	0.20 (2)
O4BA	0.6720 (3)	0.4105 (6)	0.21067 (13)	0.149 (3)	0.950 (19)
O5BA	0.8123 (3)	0.4153 (7)	0.22437 (14)	0.136 (3)	0.950 (19)
O4BB	0.6771 (15)	0.376 (6)	0.2114 (4)	0.149 (3)	0.050 (19)
O5BB	0.803 (2)	0.470 (5)	0.2232 (5)	0.136 (3)	0.050 (19)
O6BA	0.9445 (3)	0.3776 (7)	0.4123 (2)	0.114 (2)	0.756 (8)
O7BA	0.8773 (4)	0.4852 (11)	0.4686 (2)	0.120 (2)	0.756 (8)
O6BB	0.9310 (8)	0.461 (2)	0.3901 (5)	0.114 (2)	0.244 (8)
O7BB	0.8972 (14)	0.478 (4)	0.4700 (5)	0.120 (2)	0.244 (8)
N1B	0.5528 (2)	0.4924 (4)	0.38306 (12)	0.0794 (9)	
N2B	0.7398 (3)	0.4172 (3)	0.24045 (11)	0.1022 (17)	
N3B	0.8814 (2)	0.4406 (4)	0.42404 (12)	0.0837 (10)	
C1B	0.7204 (3)	0.4642 (3)	0.40885 (12)	0.0632 (9)	
C2B	0.6469 (3)	0.4639 (3)	0.36980 (15)	0.0606 (8)	
C3B	0.6514 (3)	0.4471 (3)	0.31648 (14)	0.0619 (9)	
H3BA	0.5999	0.4472	0.2930	0.074*	
C4B	0.7305 (3)	0.4302 (3)	0.29740 (11)	0.0612 (9)	
C5B	0.8079 (3)	0.4290 (3)	0.33118 (17)	0.0669 (10)	
H5BA	0.8623	0.4172	0.3179	0.080*	
C6B	0.8022 (3)	0.4458 (3)	0.38576 (16)	0.0669 (10)	

### Atomic displacement parameters (Å^2^)

**Table:**

	*U*^11^	*U*^22^	*U*^33^	*U*^12^	*U*^13^	*U*^23^
N1	0.0331 (10)	0.0443 (11)	0.0259 (9)	0.0034 (8)	0.0041 (7)	0.0004 (8)
N2	0.0504 (12)	0.0432 (12)	0.0302 (10)	0.0019 (9)	0.0047 (9)	−0.0040 (9)
C1	0.0331 (12)	0.0481 (14)	0.0418 (13)	0.0051 (10)	0.0076 (10)	−0.0005 (11)
C2	0.0414 (13)	0.0484 (15)	0.0393 (13)	0.0070 (11)	−0.0001 (10)	0.0017 (11)
C3	0.0405 (13)	0.0501 (15)	0.0391 (13)	−0.0047 (11)	0.0063 (10)	−0.0046 (11)
C4	0.0356 (12)	0.0506 (14)	0.0339 (12)	0.0023 (11)	0.0002 (9)	−0.0012 (10)
C5	0.0480 (13)	0.0452 (13)	0.0225 (10)	0.0037 (11)	0.0057 (9)	0.0033 (9)
C6	0.0417 (13)	0.0409 (13)	0.0349 (12)	0.0006 (10)	0.0003 (10)	0.0013 (10)
C7	0.0501 (15)	0.0670 (19)	0.0396 (14)	0.0057 (14)	−0.0002 (11)	0.0096 (13)
C8	0.0548 (18)	0.077 (2)	0.066 (2)	0.0148 (17)	−0.0096 (15)	0.0207 (18)
C9	0.0429 (15)	0.0615 (19)	0.079 (2)	0.0129 (14)	−0.0023 (14)	−0.0033 (16)
C10	0.0477 (16)	0.068 (2)	0.0591 (18)	0.0118 (14)	0.0060 (13)	−0.0144 (15)
C11	0.0516 (15)	0.0595 (17)	0.0372 (14)	0.0122 (13)	0.0006 (11)	−0.0033 (12)
C12	0.0407 (13)	0.0485 (14)	0.0395 (13)	0.0050 (11)	0.0096 (10)	0.0054 (11)
C13	0.0509 (17)	0.100 (3)	0.0545 (19)	−0.0023 (18)	0.0131 (14)	0.0241 (18)
C14	0.0514 (19)	0.111 (4)	0.102 (3)	−0.015 (2)	0.021 (2)	0.041 (3)
C15	0.0446 (17)	0.083 (3)	0.113 (3)	−0.0171 (17)	−0.0048 (19)	0.018 (2)
C16	0.0619 (19)	0.070 (2)	0.068 (2)	−0.0113 (17)	−0.0049 (16)	−0.0060 (17)
C17	0.0530 (16)	0.0623 (18)	0.0419 (15)	−0.0087 (14)	0.0042 (12)	0.0006 (13)
C18A	0.079 (2)	0.0422 (15)	0.0474 (16)	0.0015 (14)	0.0080 (14)	0.0001 (12)
C19A	0.058 (3)	0.049 (2)	0.053 (2)	0.000 (3)	0.011 (3)	0.0089 (18)
C20A	0.060 (2)	0.050 (2)	0.047 (2)	0.004 (2)	0.012 (2)	0.0043 (18)
C21A	0.060 (7)	0.039 (3)	0.063 (5)	0.003 (6)	0.021 (5)	0.004 (3)
C22A	0.043 (6)	0.059 (4)	0.069 (7)	0.000 (5)	−0.011 (4)	0.008 (5)
C23A	0.062 (6)	0.056 (8)	0.078 (9)	0.006 (5)	−0.001 (5)	−0.005 (7)
C24A	0.115 (15)	0.042 (6)	0.046 (8)	−0.021 (7)	−0.001 (7)	−0.003 (4)
C25A	0.085 (12)	0.065 (10)	0.088 (14)	−0.001 (7)	0.021 (8)	−0.023 (7)
C26A	0.071 (11)	0.050 (5)	0.084 (10)	−0.001 (7)	−0.040 (9)	−0.001 (5)
C18B	0.079 (2)	0.0422 (15)	0.0474 (16)	0.0015 (14)	0.0080 (14)	0.0001 (12)
C19B	0.058 (3)	0.049 (2)	0.053 (2)	0.000 (3)	0.011 (3)	0.0089 (18)
C20B	0.060 (2)	0.050 (2)	0.047 (2)	0.004 (2)	0.012 (2)	0.0043 (18)
C21B	0.052 (8)	0.041 (4)	0.043 (5)	0.003 (7)	0.020 (5)	0.003 (3)
C22B	0.026 (7)	0.067 (6)	0.054 (8)	0.002 (6)	−0.017 (5)	0.004 (6)
C23B	0.13 (3)	0.066 (16)	0.11 (2)	−0.007 (13)	−0.018 (18)	0.042 (15)
C24B	0.11 (2)	0.070 (12)	0.030 (8)	0.046 (11)	0.019 (9)	0.004 (6)
C25B	0.080 (12)	0.037 (7)	0.037 (8)	0.006 (7)	0.019 (7)	0.003 (6)
C26B	0.064 (12)	0.042 (5)	0.042 (6)	0.004 (8)	−0.026 (8)	−0.003 (4)
O1A	0.111 (2)	0.0770 (16)	0.0255 (10)	−0.0050 (14)	0.0153 (11)	0.0045 (10)
O2AA	0.092 (5)	0.157 (7)	0.068 (2)	0.028 (5)	−0.035 (2)	−0.036 (3)
O3AA	0.036 (3)	0.371 (16)	0.098 (3)	0.009 (6)	−0.004 (3)	−0.030 (7)
O2AB	0.092 (5)	0.157 (7)	0.068 (2)	0.028 (5)	−0.035 (2)	−0.036 (3)
O3AB	0.036 (3)	0.371 (16)	0.098 (3)	0.009 (6)	−0.004 (3)	−0.030 (7)
O4A	0.112 (2)	0.102 (2)	0.0538 (15)	−0.0046 (18)	0.0436 (15)	0.0099 (14)
O5A	0.118 (2)	0.095 (2)	0.0289 (11)	0.0025 (17)	0.0035 (13)	0.0106 (11)
O6AA	0.062 (2)	0.271 (8)	0.068 (3)	−0.027 (3)	−0.0050 (18)	0.040 (4)
O7AA	0.090 (3)	0.265 (9)	0.083 (4)	−0.038 (4)	0.050 (3)	−0.053 (5)
O6AB	0.062 (2)	0.271 (8)	0.068 (3)	−0.027 (3)	−0.0050 (18)	0.040 (4)
O7AB	0.090 (3)	0.265 (9)	0.083 (4)	−0.038 (4)	0.050 (3)	−0.053 (5)
N1A	0.0604 (17)	0.090 (2)	0.0574 (17)	0.0002 (16)	−0.0077 (13)	−0.0144 (15)
N2A	0.096 (2)	0.0496 (14)	0.0352 (13)	0.0009 (14)	0.0206 (14)	0.0022 (10)
N3A	0.0589 (17)	0.114 (3)	0.0562 (17)	−0.0070 (18)	0.0158 (14)	0.0052 (17)
C1A	0.0646 (17)	0.0379 (13)	0.0272 (12)	−0.0032 (12)	0.0059 (11)	−0.0032 (9)
C2A	0.0566 (16)	0.0450 (14)	0.0361 (13)	−0.0022 (12)	−0.0001 (11)	−0.0035 (11)
C3A	0.0599 (17)	0.0450 (14)	0.0453 (15)	−0.0080 (13)	0.0163 (12)	−0.0041 (12)
C4A	0.0696 (18)	0.0384 (13)	0.0291 (13)	−0.0002 (12)	0.0115 (11)	0.0030 (10)
C5A	0.0610 (17)	0.0491 (15)	0.0330 (13)	0.0019 (13)	0.0029 (11)	0.0031 (11)
C6A	0.0553 (16)	0.0532 (16)	0.0349 (13)	−0.0051 (13)	0.0101 (11)	−0.0004 (11)
O1B	0.0833 (19)	0.145 (3)	0.0486 (14)	−0.0058 (19)	0.0058 (12)	−0.0186 (16)
O2BA	0.047 (4)	0.159 (5)	0.123 (3)	−0.007 (3)	0.023 (2)	0.013 (3)
O3BA	0.083 (3)	0.166 (6)	0.126 (4)	0.036 (3)	−0.002 (3)	0.028 (5)
O2BB	0.047 (4)	0.159 (5)	0.123 (3)	−0.007 (3)	0.023 (2)	0.013 (3)
O3BB	0.083 (3)	0.166 (6)	0.126 (4)	0.036 (3)	−0.002 (3)	0.028 (5)
O4BA	0.321 (8)	0.073 (3)	0.0417 (16)	0.015 (3)	−0.037 (3)	−0.0044 (15)
O5BA	0.267 (6)	0.079 (4)	0.081 (2)	0.013 (3)	0.109 (4)	0.0066 (18)
O4BB	0.321 (8)	0.073 (3)	0.0417 (16)	0.015 (3)	−0.037 (3)	−0.0044 (15)
O5BB	0.267 (6)	0.079 (4)	0.081 (2)	0.013 (3)	0.109 (4)	0.0066 (18)
O6BA	0.060 (2)	0.199 (7)	0.083 (3)	0.038 (3)	0.002 (2)	−0.019 (3)
O7BA	0.069 (4)	0.198 (5)	0.087 (2)	0.029 (4)	−0.015 (2)	−0.044 (3)
O6BB	0.060 (2)	0.199 (7)	0.083 (3)	0.038 (3)	0.002 (2)	−0.019 (3)
O7BB	0.069 (4)	0.198 (5)	0.087 (2)	0.029 (4)	−0.015 (2)	−0.044 (3)
N1B	0.086 (2)	0.075 (2)	0.077 (2)	−0.0108 (18)	0.0077 (19)	0.0077 (17)
N2B	0.219 (6)	0.0467 (17)	0.0441 (18)	0.013 (2)	0.030 (3)	0.0043 (14)
N3B	0.068 (2)	0.104 (3)	0.079 (2)	−0.0047 (19)	0.0071 (17)	−0.016 (2)
C1B	0.106 (3)	0.0482 (16)	0.0361 (15)	−0.0035 (17)	0.0081 (16)	−0.0068 (12)
C2B	0.078 (2)	0.0448 (16)	0.0622 (19)	−0.0044 (15)	0.0237 (17)	−0.0017 (14)
C3B	0.085 (2)	0.0398 (15)	0.0569 (19)	−0.0046 (15)	−0.0138 (17)	−0.0028 (13)
C4B	0.115 (3)	0.0375 (14)	0.0333 (14)	0.0022 (16)	0.0168 (16)	0.0006 (11)
C5B	0.077 (2)	0.0393 (15)	0.089 (3)	0.0016 (15)	0.031 (2)	0.0018 (16)
C6B	0.077 (2)	0.0464 (16)	0.072 (2)	−0.0066 (16)	−0.0183 (18)	0.0004 (15)

### Geometric parameters (Å, °)

**Table:**

N1—C4	1.505 (3)	C19B—C20B	1.309 (12)
N1—C1	1.505 (3)	C19B—H19B	0.9300
N1—C5	1.522 (3)	C20B—C21B	1.493 (9)
N1—H1N	0.901 (5)	C20B—H20B	0.9300
N2—C2	1.488 (4)	C21B—C26B	1.30 (4)
N2—C3	1.490 (4)	C21B—C22B	1.385 (14)
N2—C18A	1.524 (4)	C22B—C23B	1.47 (5)
N2—H2N	0.897 (5)	C22B—H22B	0.9300
C1—C2	1.514 (4)	C23B—C24B	1.35 (5)
C1—H1A	0.9700	C23B—H23B	0.9300
C1—H1B	0.9700	C24B—C25B	1.40 (6)
C2—H2A	0.9700	C24B—H24B	0.9300
C2—H2B	0.9700	C25B—C26B	1.39 (4)
C3—C4	1.515 (4)	C25B—H25B	0.9300
C3—H3A	0.9700	C26B—H26B	0.9300
C3—H3B	0.9700	O1A—C1A	1.241 (3)
C4—H4A	0.9700	O2AA—N1A	1.2115 (16)
C4—H4B	0.9700	O3AA—N1A	1.2120 (17)
C5—C6	1.513 (4)	O2AB—N1A	1.2115 (17)
C5—C12	1.513 (4)	O3AB—N1A	1.2118 (17)
C5—H5A	0.9800	O4A—N2A	1.2118 (16)
C6—C7	1.382 (4)	O5A—N2A	1.2125 (16)
C6—C11	1.389 (4)	O6AA—N3A	1.2122 (16)
C7—C8	1.381 (5)	O7AA—N3A	1.2115 (16)
C7—H7A	0.9300	O6AB—N3A	1.2117 (17)
C8—C9	1.367 (6)	O7AB—N3A	1.2117 (17)
C8—H8A	0.9300	N1A—C2A	1.461 (4)
C9—C10	1.375 (5)	N2A—C4A	1.446 (3)
C9—H9A	0.9300	N3A—C6A	1.433 (4)
C10—C11	1.378 (4)	C1A—C2A	1.432 (4)
C10—H10A	0.9300	C1A—C6A	1.443 (4)
C11—H11A	0.9300	C2A—C3A	1.388 (4)
C12—C13	1.388 (4)	C3A—C4A	1.370 (4)
C12—C17	1.394 (4)	C3A—H3AA	0.9300
C13—C14	1.386 (6)	C4A—C5A	1.377 (4)
C13—H13A	0.9300	C5A—C6A	1.371 (4)
C14—C15	1.371 (7)	C5A—H5AA	0.9300
C14—H14A	0.9300	O1B—C1B	1.259 (4)
C15—C16	1.374 (6)	O2BA—N1B	1.2101 (17)
C15—H15A	0.9300	O3BA—N1B	1.2111 (17)
C16—C17	1.380 (5)	O2BB—N1B	1.2118 (17)
C16—H16A	0.9300	O3BB—N1B	1.2116 (17)
C17—H17A	0.9300	O4BA—N2B	1.2109 (16)
C18A—C19A	1.546 (7)	O5BA—N2B	1.2120 (16)
C18A—H18A	0.9700	O4BB—N2B	1.2119 (17)
C18A—H18B	0.9700	O5BB—N2B	1.2119 (17)
C19A—C20A	1.310 (9)	O6BA—N3B	1.2132 (16)
C19A—H19A	0.9300	O7BA—N3B	1.2120 (16)
C20A—C21A	1.483 (8)	O6BB—N3B	1.2116 (17)
C20A—H20A	0.9300	O7BB—N3B	1.2117 (17)
C21A—C22A	1.382 (12)	N1B—C2B	1.528 (5)
C21A—C26A	1.46 (3)	N2B—C4B	1.455 (4)
C22A—C23A	1.29 (3)	N3B—C6B	1.461 (5)
C22A—H22A	0.9300	C1B—C2B	1.407 (6)
C23A—C24A	1.33 (3)	C1B—C6B	1.437 (6)
C23A—H23A	0.9300	C2B—C3B	1.356 (5)
C24A—C25A	1.36 (5)	C3B—C4B	1.350 (6)
C24A—H24A	0.9300	C3B—H3BA	0.9300
C25A—C26A	1.42 (4)	C4B—C5B	1.376 (6)
C25A—H25A	0.9300	C5B—C6B	1.390 (6)
C26A—H26A	0.9300	C5B—H5BA	0.9300
			
Cg1···Cg3	3.844 (7)	Cg2···Cg4	3.634 (2)
Cg1···Cg4	3.677 (9)	Cg3···Cg4^i^	3.729 (7)
Cg2···Cg3	3.825 (5)		
			
C4—N1—C1	108.3 (2)	C25A—C26A—C21A	127.5 (18)
C4—N1—C5	111.38 (19)	C25A—C26A—H26A	116.3
C1—N1—C5	110.63 (19)	C21A—C26A—H26A	116.3
C4—N1—H1N	107 (2)	C20B—C19B—H19B	120.2
C1—N1—H1N	107 (2)	C19B—C20B—C21B	124.0 (9)
C5—N1—H1N	113 (2)	C19B—C20B—H20B	118.0
C2—N2—C3	110.4 (2)	C21B—C20B—H20B	118.0
C2—N2—C18A	112.0 (2)	C26B—C21B—C22B	121.5 (13)
C3—N2—C18A	111.5 (2)	C26B—C21B—C20B	110.3 (13)
C2—N2—H2N	103 (3)	C22B—C21B—C20B	128.1 (12)
C3—N2—H2N	114 (3)	C21B—C22B—C23B	120 (2)
C18A—N2—H2N	106 (3)	C21B—C22B—H22B	120.2
N1—C1—C2	110.7 (2)	C23B—C22B—H22B	120.3
N1—C1—H1A	109.5	C24B—C23B—C22B	123 (4)
C2—C1—H1A	109.5	C24B—C23B—H23B	118.5
N1—C1—H1B	109.5	C22B—C23B—H23B	118.5
C2—C1—H1B	109.5	C23B—C24B—C25B	108 (3)
H1A—C1—H1B	108.1	C23B—C24B—H24B	126.1
N2—C2—C1	111.4 (2)	C25B—C24B—H24B	126.1
N2—C2—H2A	109.3	C26B—C25B—C24B	134 (3)
C1—C2—H2A	109.3	C26B—C25B—H25B	113.1
N2—C2—H2B	109.3	C24B—C25B—H25B	113.1
C1—C2—H2B	109.3	C21B—C26B—C25B	114 (2)
H2A—C2—H2B	108.0	C21B—C26B—H26B	123.1
N2—C3—C4	111.1 (2)	C25B—C26B—H26B	123.1
N2—C3—H3A	109.4	O2AB—N1A—O3AB	122.6 (2)
C4—C3—H3A	109.4	O2AA—N1A—O3AB	120.6 (19)
N2—C3—H3B	109.4	O2AB—N1A—O3AA	111.7 (16)
C4—C3—H3B	109.4	O2AA—N1A—O3AA	122.6 (2)
H3A—C3—H3B	108.0	O2AB—N1A—C2A	125.8 (12)
N1—C4—C3	110.8 (2)	O2AA—N1A—C2A	117.5 (5)
N1—C4—H4A	109.5	O3AB—N1A—C2A	109.6 (10)
C3—C4—H4A	109.5	O3AA—N1A—C2A	119.9 (5)
N1—C4—H4B	109.5	O4A—N2A—O5A	122.6 (2)
C3—C4—H4B	109.5	O4A—N2A—C4A	118.4 (2)
H4A—C4—H4B	108.1	O5A—N2A—C4A	119.0 (2)
C6—C5—C12	110.6 (2)	O7AA—N3A—O6AB	106.6 (14)
C6—C5—N1	112.2 (2)	O7AB—N3A—O6AB	122.6 (2)
C12—C5—N1	112.0 (2)	O7AA—N3A—O6AA	122.6 (2)
C6—C5—H5A	107.2	O7AB—N3A—O6AA	114 (2)
C12—C5—H5A	107.2	O7AA—N3A—C6A	120.1 (3)
N1—C5—H5A	107.2	O7AB—N3A—C6A	116.2 (13)
C7—C6—C11	118.3 (3)	O6AB—N3A—C6A	121.2 (13)
C7—C6—C5	119.1 (2)	O6AA—N3A—C6A	117.4 (3)
C11—C6—C5	122.3 (2)	O1A—C1A—C2A	124.6 (3)
C8—C7—C6	120.8 (3)	O1A—C1A—C6A	123.3 (3)
C8—C7—H7A	119.6	C2A—C1A—C6A	112.1 (2)
C6—C7—H7A	119.6	C3A—C2A—C1A	124.4 (3)
C9—C8—C7	120.2 (3)	C3A—C2A—N1A	116.3 (3)
C9—C8—H8A	119.9	C1A—C2A—N1A	119.3 (2)
C7—C8—H8A	119.9	C4A—C3A—C2A	118.7 (3)
C8—C9—C10	119.7 (3)	C4A—C3A—H3AA	120.7
C8—C9—H9A	120.1	C2A—C3A—H3AA	120.7
C10—C9—H9A	120.1	C3A—C4A—C5A	121.4 (2)
C9—C10—C11	120.4 (3)	C3A—C4A—N2A	120.2 (3)
C9—C10—H10A	119.8	C5A—C4A—N2A	118.4 (3)
C11—C10—H10A	119.8	C6A—C5A—C4A	119.4 (3)
C10—C11—C6	120.5 (3)	C6A—C5A—H5AA	120.3
C10—C11—H11A	119.8	C4A—C5A—H5AA	120.3
C6—C11—H11A	119.8	C5A—C6A—N3A	116.3 (3)
C13—C12—C17	117.9 (3)	C5A—C6A—C1A	124.0 (3)
C13—C12—C5	119.1 (3)	N3A—C6A—C1A	119.7 (2)
C17—C12—C5	122.8 (2)	O2BA—N1B—O3BA	122.8 (2)
C14—C13—C12	120.9 (4)	O2BA—N1B—O3BB	108.0 (17)
C14—C13—H13A	119.5	O3BA—N1B—O2BB	140.1 (12)
C12—C13—H13A	119.5	O3BB—N1B—O2BB	122.6 (2)
C15—C14—C13	120.3 (3)	O2BA—N1B—C2B	119.9 (4)
C15—C14—H14A	119.8	O3BA—N1B—C2B	117.2 (5)
C13—C14—H14A	119.8	O3BB—N1B—C2B	131.8 (18)
C14—C15—C16	119.5 (4)	O2BB—N1B—C2B	100.3 (10)
C14—C15—H15A	120.2	O4BA—N2B—O5BB	117.3 (15)
C16—C15—H15A	120.2	O4BB—N2B—O5BB	122.6 (2)
C15—C16—C17	120.7 (4)	O4BA—N2B—O5BA	122.6 (2)
C15—C16—H16A	119.6	O4BB—N2B—O5BA	118.7 (11)
C17—C16—H16A	119.6	O4BA—N2B—C4B	116.6 (3)
C16—C17—C12	120.6 (3)	O4BB—N2B—C4B	118.0 (3)
C16—C17—H17A	119.7	O5BB—N2B—C4B	118.0 (3)
C12—C17—H17A	119.7	O5BA—N2B—C4B	120.7 (3)
N2—C18A—C19A	112.8 (3)	O6BB—N3B—O7BB	122.6 (3)
N2—C18A—H18A	109.0	O6BB—N3B—O7BA	132.8 (12)
C19A—C18A—H18A	109.0	O7BB—N3B—O6BA	107.9 (17)
N2—C18A—H18B	109.0	O7BA—N3B—O6BA	122.4 (2)
C19A—C18A—H18B	109.0	O6BB—N3B—C6B	93.6 (8)
H18A—C18A—H18B	107.8	O7BB—N3B—C6B	133.1 (15)
C20A—C19A—C18A	121.1 (5)	O7BA—N3B—C6B	118.4 (3)
C20A—C19A—H19A	119.4	O6BA—N3B—C6B	118.6 (3)
C18A—C19A—H19A	119.4	O1B—C1B—C2B	117.7 (4)
C19A—C20A—C21A	129.3 (7)	O1B—C1B—C6B	130.2 (4)
C19A—C20A—H20A	115.4	C2B—C1B—C6B	112.1 (3)
C21A—C20A—H20A	115.4	C3B—C2B—C1B	124.7 (4)
C22A—C21A—C26A	114.1 (13)	C3B—C2B—N1B	112.4 (3)
C22A—C21A—C20A	121.5 (10)	C1B—C2B—N1B	122.8 (3)
C26A—C21A—C20A	124.4 (14)	C4B—C3B—C2B	120.1 (3)
C23A—C22A—C21A	119.1 (14)	C4B—C3B—H3BA	119.9
C23A—C22A—H22A	120.4	C2B—C3B—H3BA	119.9
C21A—C22A—H22A	120.4	C3B—C4B—C5B	121.2 (3)
C22A—C23A—C24A	124 (2)	C3B—C4B—N2B	122.6 (4)
C22A—C23A—H23A	117.9	C5B—C4B—N2B	116.1 (4)
C24A—C23A—H23A	117.9	C4B—C5B—C6B	118.0 (3)
C23A—C24A—C25A	128 (2)	C4B—C5B—H5BA	121.0
C23A—C24A—H24A	115.9	C6B—C5B—H5BA	121.0
C25A—C24A—H24A	115.9	C5B—C6B—C1B	123.8 (3)
C24A—C25A—C26A	106 (2)	C5B—C6B—N3B	120.8 (4)
C24A—C25A—H25A	126.8	C1B—C6B—N3B	115.4 (3)
C26A—C25A—H25A	126.8		
			
C4—N1—C1—C2	−58.5 (3)	O3AA—N1A—C2A—C3A	14.3 (14)
C5—N1—C1—C2	179.1 (2)	O2AB—N1A—C2A—C1A	37 (2)
C3—N2—C2—C1	−55.7 (3)	O2AA—N1A—C2A—C1A	15.2 (9)
C18A—N2—C2—C1	179.5 (2)	O3AB—N1A—C2A—C1A	−127.4 (18)
N1—C1—C2—N2	58.0 (3)	O3AA—N1A—C2A—C1A	−163.3 (13)
C2—N2—C3—C4	55.7 (3)	C1A—C2A—C3A—C4A	−0.9 (4)
C18A—N2—C3—C4	−179.2 (2)	N1A—C2A—C3A—C4A	−178.3 (3)
C1—N1—C4—C3	58.8 (3)	C2A—C3A—C4A—C5A	0.4 (4)
C5—N1—C4—C3	−179.36 (19)	C2A—C3A—C4A—N2A	179.5 (3)
N2—C3—C4—N1	−58.3 (3)	O4A—N2A—C4A—C3A	4.0 (4)
C4—N1—C5—C6	54.6 (3)	O5A—N2A—C4A—C3A	−176.3 (3)
C1—N1—C5—C6	175.1 (2)	O4A—N2A—C4A—C5A	−176.9 (3)
C4—N1—C5—C12	179.7 (2)	O5A—N2A—C4A—C5A	2.8 (4)
C1—N1—C5—C12	−59.8 (3)	C3A—C4A—C5A—C6A	−0.5 (5)
C12—C5—C6—C7	104.0 (3)	N2A—C4A—C5A—C6A	−179.6 (3)
N1—C5—C6—C7	−130.2 (3)	C4A—C5A—C6A—N3A	179.0 (3)
C12—C5—C6—C11	−70.3 (3)	C4A—C5A—C6A—C1A	1.1 (5)
N1—C5—C6—C11	55.6 (3)	O7AA—N3A—C6A—C5A	160.3 (7)
C11—C6—C7—C8	1.6 (5)	O7AB—N3A—C6A—C5A	119 (3)
C5—C6—C7—C8	−172.9 (3)	O6AB—N3A—C6A—C5A	−62 (2)
C6—C7—C8—C9	−0.7 (6)	O6AA—N3A—C6A—C5A	−20.9 (7)
C7—C8—C9—C10	−1.4 (6)	O7AA—N3A—C6A—C1A	−21.7 (8)
C8—C9—C10—C11	2.6 (6)	O7AB—N3A—C6A—C1A	−63 (3)
C9—C10—C11—C6	−1.6 (5)	O6AB—N3A—C6A—C1A	116 (2)
C7—C6—C11—C10	−0.4 (5)	O6AA—N3A—C6A—C1A	157.1 (6)
C5—C6—C11—C10	173.9 (3)	O1A—C1A—C6A—C5A	176.1 (3)
C6—C5—C12—C13	−95.4 (3)	C2A—C1A—C6A—C5A	−1.4 (4)
N1—C5—C12—C13	138.6 (3)	O1A—C1A—C6A—N3A	−1.7 (5)
C6—C5—C12—C17	78.4 (3)	C2A—C1A—C6A—N3A	−179.3 (3)
N1—C5—C12—C17	−47.6 (3)	O1B—C1B—C2B—C3B	179.4 (4)
C17—C12—C13—C14	−1.2 (5)	C6B—C1B—C2B—C3B	0.6 (5)
C5—C12—C13—C14	172.9 (4)	O1B—C1B—C2B—N1B	3.7 (5)
C12—C13—C14—C15	1.7 (7)	C6B—C1B—C2B—N1B	−175.1 (3)
C13—C14—C15—C16	−0.4 (7)	O2BA—N1B—C2B—C3B	151.3 (6)
C14—C15—C16—C17	−1.3 (7)	O3BA—N1B—C2B—C3B	−24.4 (10)
C15—C16—C17—C12	1.7 (6)	O3BB—N1B—C2B—C3B	−37 (4)
C13—C12—C17—C16	−0.5 (5)	O2BB—N1B—C2B—C3B	169.8 (18)
C5—C12—C17—C16	−174.3 (3)	O2BA—N1B—C2B—C1B	−32.6 (7)
C2—N2—C18A—C19A	74.9 (4)	O3BA—N1B—C2B—C1B	151.8 (9)
C3—N2—C18A—C19A	−49.4 (4)	O3BB—N1B—C2B—C1B	139 (4)
N2—C18A—C19A—C20A	−100.0 (5)	O2BB—N1B—C2B—C1B	−14.1 (19)
C18A—C19A—C20A—C21A	−178.2 (7)	C1B—C2B—C3B—C4B	−0.6 (5)
C19A—C20A—C21A—C22A	−176.6 (8)	N1B—C2B—C3B—C4B	175.5 (3)
C19A—C20A—C21A—C26A	4(2)	C2B—C3B—C4B—C5B	0.3 (5)
C26A—C21A—C22A—C23A	2(2)	C2B—C3B—C4B—N2B	−177.4 (3)
C20A—C21A—C22A—C23A	−177.1 (14)	O4BA—N2B—C4B—C3B	−6.4 (6)
C21A—C22A—C23A—C24A	1(3)	O4BB—N2B—C4B—C3B	−25 (3)
C22A—C23A—C24A—C25A	−7(3)	O5BB—N2B—C4B—C3B	142 (3)
C23A—C24A—C25A—C26A	8(3)	O5BA—N2B—C4B—C3B	172.7 (5)
C24A—C25A—C26A—C21A	−5(4)	O4BA—N2B—C4B—C5B	175.9 (4)
C22A—C21A—C26A—C25A	1(3)	O4BB—N2B—C4B—C5B	157 (3)
C20A—C21A—C26A—C25A	180 (2)	O5BB—N2B—C4B—C5B	−36 (3)
C19B—C20B—C21B—C26B	178.7 (18)	O5BA—N2B—C4B—C5B	−5.1 (6)
C19B—C20B—C21B—C22B	−5(2)	C3B—C4B—C5B—C6B	−0.1 (5)
C26B—C21B—C22B—C23B	−2(3)	N2B—C4B—C5B—C6B	177.7 (3)
C20B—C21B—C22B—C23B	−178 (2)	C4B—C5B—C6B—C1B	0.1 (5)
C21B—C22B—C23B—C24B	0(4)	C4B—C5B—C6B—N3B	177.7 (3)
C22B—C23B—C24B—C25B	4(4)	O1B—C1B—C6B—C5B	−178.9 (4)
C23B—C24B—C25B—C26B	−8(5)	C2B—C1B—C6B—C5B	−0.3 (5)
C22B—C21B—C26B—C25B	−1(4)	O1B—C1B—C6B—N3B	3.4 (6)
C20B—C21B—C26B—C25B	175 (2)	C2B—C1B—C6B—N3B	−178.0 (3)
C24B—C25B—C26B—C21B	7(5)	O6BB—N3B—C6B—C5B	19.3 (11)
O1A—C1A—C2A—C3A	−176.2 (3)	O7BB—N3B—C6B—C5B	162 (2)
C6A—C1A—C2A—C3A	1.3 (4)	O7BA—N3B—C6B—C5B	163.2 (7)
O1A—C1A—C2A—N1A	1.1 (5)	O6BA—N3B—C6B—C5B	−25.8 (7)
C6A—C1A—C2A—N1A	178.6 (3)	O6BB—N3B—C6B—C1B	−162.9 (11)
O2AB—N1A—C2A—C3A	−146 (2)	O7BB—N3B—C6B—C1B	−20 (2)
O2AA—N1A—C2A—C3A	−167.3 (8)	O7BA—N3B—C6B—C1B	−19.0 (9)
O3AB—N1A—C2A—C3A	50.1 (18)	O6BA—N3B—C6B—C1B	152.0 (5)

Symmetry codes:  (i) *x*, *y*+1, *z*.

### Hydrogen-bond geometry (Å, °)

**Table:**

Cg5 and Cg6 are the centroids of the C6–C11 and C12–C17 rings, respectively.

**Table:**

*D*—H···*A*	*D*—H	H···*A*	*D*···*A*	*D*—H···*A*
N1—H1N···O1A	0.90 (1)	1.77 (1)	2.658 (3)	168 (3)
N2—H2N···O1B	0.90 (1)	1.83 (3)	2.603 (3)	143 (4)
N2—H2N···O7BA	0.90 (1)	2.30 (3)	3.047 (5)	140 (3)
C3—H3A···O2AA	0.97	2.50	3.214 (8)	130
C3—H3A···O2AB	0.97	2.52	3.242 (17)	131
C2—H2B···O7AA	0.97	2.48	3.304 (5)	142
C2—H2B···O7AB	0.97	2.54	3.42 (4)	151
C11—H11A···O4BA^ii^	0.93	2.57	3.244 (4)	130
C17—H17A···O1A	0.93	2.62	3.473 (4)	154
C17—H17A···O5BB^ii^	0.93	2.52	3.267 (8)	138
C18A—H18A···Cg6^i^	0.97	2.88	3.742 (5)	148
C18A—H18B···Cg5^i^	0.97	2.83	3.762 (0)	161
C18A—H18C···Cg5^i^	0.97	3.00	3.762 (0)	137
C18A—H18D···Cg6^i^	0.97	2.84	3.742 (5)	155

Symmetry codes: (ii) *x*, −*y*+3/2, *z*+1/2; (i) *x*, *y*+1, *z*.
